# Structural basis for the dual catalytic activity of the *Legionella pneumophila* ovarian tumor (OTU) domain deubiquitinase LotA

**DOI:** 10.1016/j.jbc.2022.102414

**Published:** 2022-08-22

**Authors:** Jiwei Luo, Xinglin Ruan, Zhijie Huang, Zekai Li, Le Ye, Yongyu Wu, Xiangkai Zhen, Songying Ouyang

**Affiliations:** 1 Provincial University Key Laboratory of Cellular Stress Response and Metabolic Regulation, The Key Laboratory of Innate Immune Biology of Fujian Province, Biomedical Research Center of South China, Key Laboratory of OptoElectronic Science and Technology for Medicine of the Ministry of Education, College of Life Sciences, Fujian Normal University, Fuzhou, China; 2 Department of Neurology, Fujian Medical University Union Hospital, Fuzhou, China

**Keywords:** bacteria, dual catalytic activity, proximal and distal ubiquitin binding site, deubiquitination, ovarian tumor family deubiquitinases, K6-diUb, Legionella pneumophila, type IV secretion system, effector protein, DUB, deubiquitinase, ITC, isothermal titration calorimetry, OTU, ovarian tumor, PDB, Protein Data Bank, SAD, single-wavelength anomalous diffraction, Ub, ubiquitin, UBD, Ub-binding domain, Ub-PA, Ub-propargylamide

## Abstract

*Legionella pneumophila*, a bacterial pathogen that causes a severe pneumonia known as Legionnaires’ disease, extensively exploits the ubiquitin (Ub) pathway in the infected host cells through certain virulence effectors excreted by the Dot/Icm system. To date, several Dot/Icm effectors have been found to act as Ub ligases, and four effectors, including LotA, LotB, LotC, and Ceg7, have been identified as deubiquitinases (DUBs) from the ovarian tumor (OTU) domain family. LotA is unique among other OTU DUBs because it possesses two distinct DUB domains and exclusively exhibits catalytic activity against K6-linked diUb and polyUb chains. However, the structure of LotA and the molecular mechanism for the dual DUB activity remains elusive. In this study, we solved the structure of LotA in complex with proximally bound Ub and distal covalently bound Ub. Both Ub molecules are bound to the DUB1 domain and mimic a K6-linked diUb. Structural analysis reveals that the DUB1 domain utilizes a distinct mechanism for recognition of the K6-linked diUb within a large S1′ binding site that is uncommon to OTU DUBs. Structural fold of the LotA DUB2 domain closely resembles LotB and LotC, similarly containing an extra α-helix lobe that has been demonstrated to play an important role in Ub binding. Collectively, our study uncovers the structural basis for the dual catalytic activity of the unique OTU family DUB LotA.

Ubiquitination, that is, the covalent attachment of the small protein ubiquitin (Ub) to substrate proteins, is one of the most common posttranslational modifications in eukaryotic cells that regulates many fundamental cellular processes (1). Ub is most commonly bound to a substrate protein *via* an isopeptide bond between the ε-amino group of a substrate lysine residue and one of the seven lysine residues of Ub (K6, K11, K27, K29, K33, K48, and K63). One Ub (monoubiquitination) or a chain of Ubs (polyubiquitination) can be added to the substrate. Canonical ubiquitination is carried out in an ATP-dependent manner by a cascade involving the E1 Ub-activating enzymes, the E2 Ub-conjugating enzymes, and E3 Ub ligases.

Ubiquitination is a dynamic and reversible process so the Ub moieties can be removed from the substrate proteins by a specific class of proteases called deubiquitinating enzymes (deubiquitinases, DUBs). Mammalian genomes encode more than 100 different DUBs grouped into seven evolutionarily conserved families: the Ub-C-terminal hydrolases (UCHs), Ub-specific proteases (USPs), Machado-Joseph domain (MJD) DUBs, ovarian tumor (OTU) domain DUBs (OTU DUBs), the motif interacting with Ub (MIU)-containing novel DUB family (MINDYs), ZUFSP/ZUP1, and the Jab1/Mov34/Mpr1Pad1 N-Terminal+(MPN+) (JAMM) domain proteases (2). Except for the JAMM domain protease family, all other DUBs are cysteine proteases, which utilize either a catalytic triad (Cys, His, and Asn/Asp) or dyad (Cys and His) for the catalytic reaction (3). Members of the OTU domain DUBs exhibit high specificity for a certain type or a small subset of Ub linkages (4). For instance, human OTUD4 and OTUB1 selectively cleave K48-linked Ub chains (4), OTULIN/FAM105B specifically cleaves M1-linked Ub chains (5, 6, 7) and Cezanne and Cezanne2 show preference for K11-linked Ub chains (8).

Besides fundamental cellular processes, ubiquitination also plays important roles in host immune defense against bacterial infections (9, 10), and it is thus commonly targeted by numerous pathogenic microorganisms to avoid detection and promote cellular conditions suitable for the infection (10, 11, 12). *Legionella pneumophila*, the causative agent of Legionnaires’ disease, extensively modulates host cellular processes by secreting hundreds of virulence effectors into the host cell *via* the Dot/Icm system (13). Recent studies found that the host ubiquitination pathway can be hijacked by a variety of *L. pneumophila* effectors, including SidE and MavC that can carry out the entire process of ubiquitination on their own (14, 15). The SidE family (SdeA, SdeB, SdeC, and SidE) mediates the unconventional phosphoribosyl serine ubiquitination mechanism for modification of Rab33b associated with the endoplasmic reticulum (16) to disrupt the membrane trafficking (17). The ubiquitination of Rab33b is regulated in an unexpected manner and can be reversed by the calmodulin-dependent glutamylase SidJ (18, 19, 20, 21), or the Ub moiety can be directly removed by the DUBs DupA and DupB from the *L. pneumophila* (22, 23).

*L. pneumophila* utilizes four OTU-like DUBs, namely LotA (Lpg2248), LotB (Lpg1621/Ceg23), LotC (Lpg2529/Lem27), and Ceg7(Lpg0227) (24), which mimic the function of host DUBs to increase infectivity of the pathogens (11). Despite marginal level of sequence conservation between them and human OTUs, the 4 *L. pneumophila* OTU DUBs exhibit a conserved fold (25). In contrast to the common mechanism for Ub recognition in other OTU DUBs, an extended helical lobe between the catalytic cys-loop and the variable loop of LotB and LotC (24, 26, 27) was demonstrated to play an important role in the Ub binding; hence, LotB and LotC were concluded as novel OTU DUBs (24, 26, 27). LotA harbors two predicted OTU DUB domains, the polyUb on the *Legionella*-containing vacuole can be cleaved dependent on the C303 and the K6-linked Ub can be specifically removed *via* the C13-dependent DUB1, which makes it the first identified OTU DUB targeting K6-linked diUb (28). However, the structure of LotA remains unreported and the molecular mechanism underlying its activity mediated by the two separate catalytic domains needs to be elucidated.

In this study, we determined the crystal structure of LotA_1-542_ in complex with Ub at 2.64 Å resolution. The covalently bound Ub and the free Ub located in the distal and proximal sites of the first LotA DUB domain (DUB1 domain) were found to mimic a K6-linked diUb, which was hydrolyzed by LotA in a unique manner compared to the well-documented K6-linkage specific USP30. In addition, the structure of the second LotA DUB and cleavage assays reveal the molecular mechanism for polyUb hydrolysis. This study provides insights into the mechanism underlying the dual catalytic activity of the OTU DUB LotA.

## Results

### Overall structure of LotA_1–542_ in complex with Ub-propargylamide

**Figure 1:**
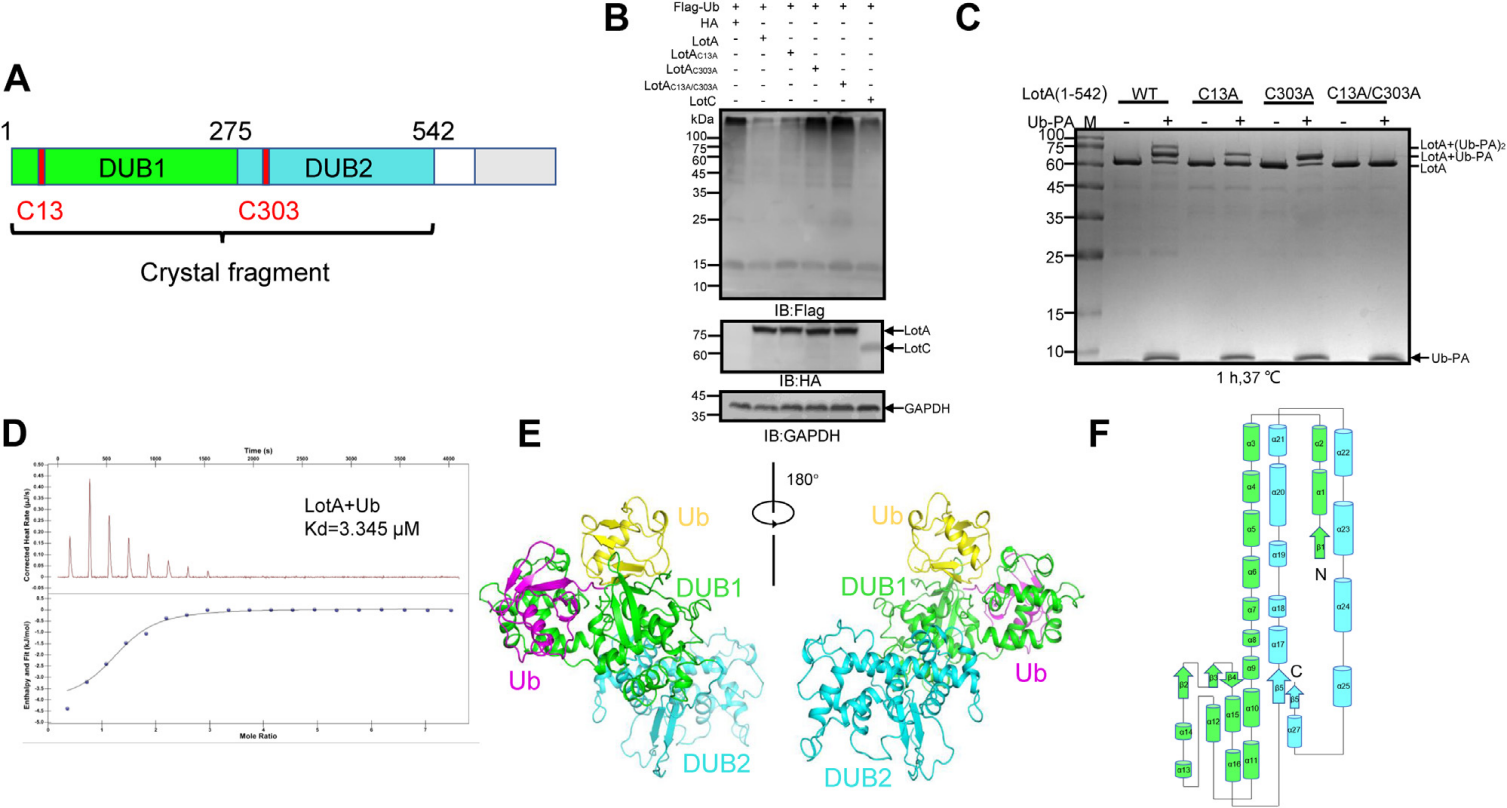
**Overall structure of LotA**_**1-542**_**with Ub-PA.***A*, domain organization of LotA. Catalytic cysteine residues within DUB1 and DUB2 domains are labeled *red*. *B*, LotA interferes with protein ubiquitination in cells. HEK293T cells were transfected to coexpress Flag-Ub and HA-LotA, HA-LotA C13A, HA-LotA C303A, HA-LotA C13A/C303A, or HA-LotC (26) as a control. Proteins modified by Flag-Ub were detected by immunoblotting with a Flag-specific antibody. The expression of LotA and its mutant variants was detected by immunoblotting using HA-specific antibodies. *C*, LotA deubiquitinase (DUB) activity *in vitro*. LotA or the catalytically inactive mutants were incubated with Ub-PA at 37 °C for 1 h and the products resolved by SDS-PAGE were detected with Coomassie blue staining. Note, the molecular weight shift of LotA after incubation with Ub-PA is observed and the molecular shift was lost when the C13, C303, or both residues were mutated to Ala. *D*, binding affinity between LotA_1-542_ and 6×His tag-labeled ubiquitin measured by ITC. The results indicate that LotA_1-542_ can bind ubiquitin with high affinity (3 μM). *E*, overall structure of LotA_1-542_ in complex with Ub-PA in the asymmetric unit (ASU) shown in two different orientations. *F*, topological map of LotA_1-520._ α-helices are represented as cylinders, and β-sheets are represented as cylinders and arrows, respectively. ITC, isothermal titration calorimetry; Ub-PA, ubiquitin-propargylamide.

To understand the structural basis for the dual DUB catalytic activity of LotA, we set out to determine the structures of apo LotA and LotA in complex with the suicide inhibitor Ub-propargylamide (Ub-PA). Full-length LotA consists of two DUB domains and a C-terminal PI3P-binding domain connected to the remainder of the protein *via* a disordered region (Fig. 1*A*). In agreement with the previous report (28), we confirmed that WT LotA, but not the inactive mutants LotA C13A, LotA C303A, and LotA C13A/C303A, can effectively remove Ub from proteins ubiquitinated by Flag-Ub (Fig. 1*B*). When selecting an appropriate LotA construct for crystallization, we considered that the disordered region is likely to hinder crystallization based on our previous experience and that the full-length LotA was found susceptible to degradation *in vitro*. Thus, the construct contained the DUB1 and DUB2 catalytic domains of LotA (amino acid residues 1–542). LotA_1-542_ was stable during expression in *Escherichia coli* and throughout the purification process, and it retained the ability to interact with Ub-PA, which can be observed as an upward shift on SDS-PAGE gel when Ub-PA is incubated with LotA_1-542_ (Fig. 1*C*). Interestingly, a ∼10 kDa band, which corresponds to Ub-PA, was always found to be coeluted with the LotA_1-542-_Ub-PA complex in size-exclusion chromatography (Fig. S1*A*). This phenomenon led us to hypothesize that Ub may specifically and noncovalently bind to LotA_1-542_ *via* an additional Ub-binding site (4), which is rarely seen in OTU DUBs. To verify this hypothesis, the binding affinity between LotA_1-542_ and Ub was measured with isothermal titration calorimetry (ITC), which indicated that LotA_1-542_ binds Ub with high affinity (3 μM) (Fig. 1*D*).

Although we failed to obtain the crystals of apo LotA_1-542_, we successfully crystallized the LotA_1-542_–Ub-PA complex and determined its structure using the single-wavelength anomalous diffraction (SAD) method (Table 1). Two LotA molecules and a total of four Ub moieties are present in the crystal asymmetric unit (Figs. 1*E* and S1). The DUB1 and DUB2 domain of each LotA moieties are similar to each other, even though certain conformational differences caused by the flexible linker were observed (Fig. S1). However, analytical ultracentrifugation shows that LotA_1-542_ has a molecular mass of 62 kDa (Fig. S1*E*), suggesting that LotA_1-542_ is a monomer in solution. The final model of LotA in the structure of LotA_1-542_–Ub-PA complex comprises residues 4 to 520. The structure reveals that both DUB1 (1–267 aa) and DUB2 (287–542 aa) exhibit a classical papain-like fold like typical OTU DUBs (4). Each DUB domain can be further divided into two subdomains. A distinctive structural feature of the DUB1 domain is the split into two compact globular α/β fold subdomains. One DUB1 globular subdomain consists of α-helices α1–α5, α13-α16, and β-sheets β1–β4, whereas the other is composed solely of helices α6–α12. Residues 287 to 520 in the DUB2 domain fold into a finger-shaped structure that is divided into two subdomains by the helix α20 (Fig. 1*F*).

**Table 1:** X-ray data collection and refinement statistics

Dataset	
Data collection	
Resolution rang (Å)	63.04–2.64 (2.734–2.64)
Wavelength (Å)	0.9792
Total Reflection	56,873 (5599)
Space group	P 2_1_ 2_1_ 2_1_
Cell dimensions	
a, b, c (Å)	73.65, 126.09, 207.09
α, β, γ (°)	90.0, 90.0, 90.0
Rmerge	0.102 (1.19)
CC1/2	0.999 (0.836)
I/σ(I)	18.3 (2.2)
Completeness (%)	99.1 (99.4)
Multiplicity	13.3 (13.8)
Refinement	
Resolution (Å)	
Rwork (%)	23.35 (34.68)
Rfree (%)	28.07 (41.21)
Ramachandran plot (%)	
Ramachandran favored (%)	96.13
Ramachandran allowed (%)	3.87
Ramachandran outliers (%)	0.00

One crystal was used for determination of each structure. Values in parentheses are for highest resolution shell.

Dali search (29) against structures in the Protein Data Bank (PDB) indicates that the structure of LotA DUB1 domain shares similarity with the OTU family DUBs, including LotB (27), LotC, and wMelOTU (30), with a Z score of 10.9 (RMSD value of 5.1 for 207 aligned residues), 9.5 (RMSD value of 4.3 Å for 197 aligned residues), and 7.5 (RMSD value of 3.0 for 164 aligned residues), respectively (Table S1). LotA DUB2 domain also shares similarity with the typical OTU DUBs, especially LotB (27), and wMelOTU with a Z score of 9.5 (RMSD value of 4.2 for 207 aligned residues) and 4.9 (RMSD value of 3.5 for 164 aligned residues), respectively (Table S2). Notably, in the complex structure, DUB1 domain binds two Ub moieties within a cleft formed between the two subdomains of DUB1, whereas no Ub was observed to be bound to the DUB2 domain despite its ability to react with Ub-PA like other cysteine-dependent DUBs (Fig. 1*C*).

### Proximally and distally bound Ubs in LotA DUB1 domain mimic a K6-linked diUb

**Figure 2:**
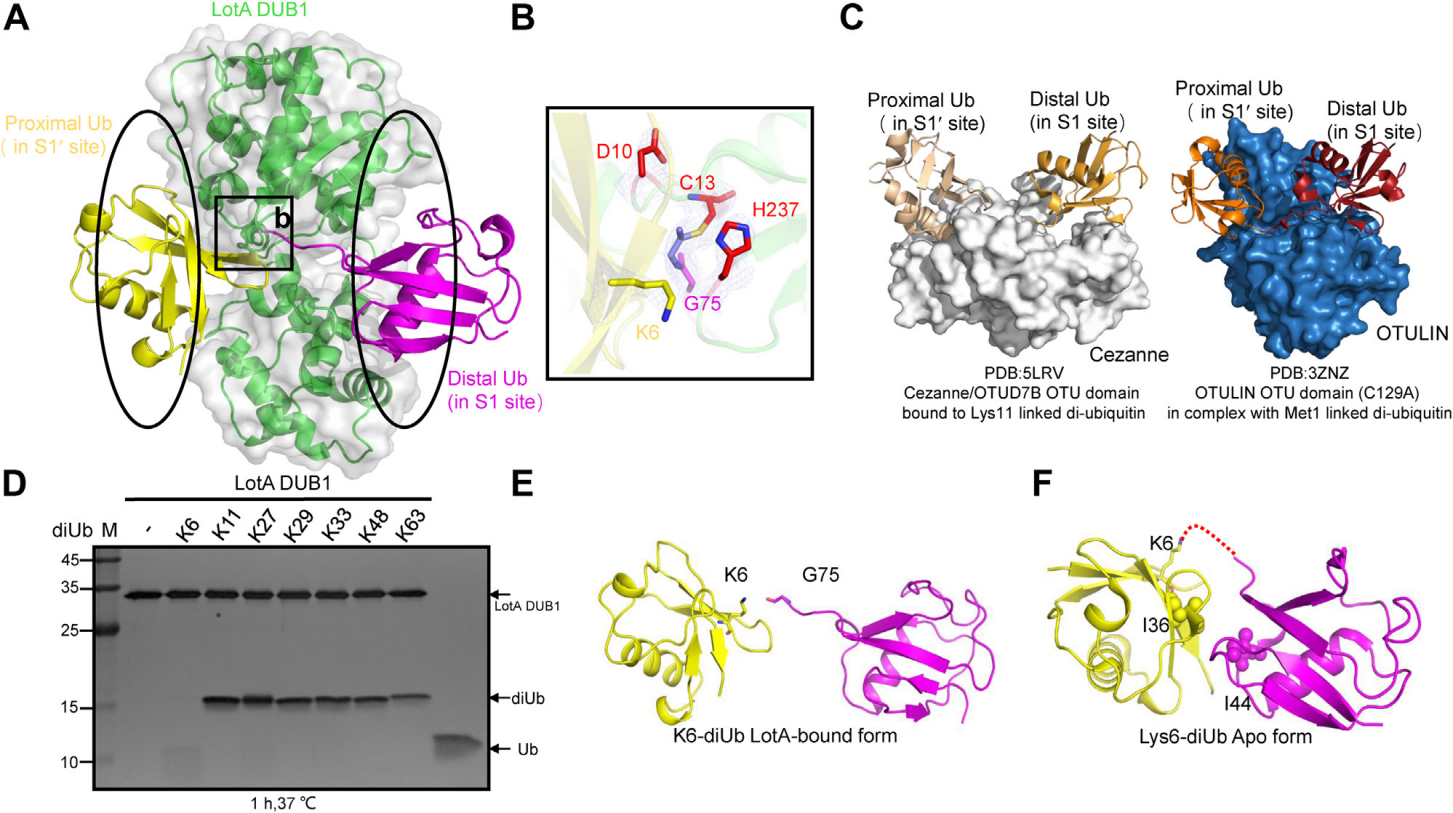
**DUB1 domain of LotA possesses a distinctive ubiquitin-binding site S1′ to ensure the accommodation of the proximal ubiquitin that mimics a K6-linked diUb together with the distally bound ubiquitin.***A*, mixed cartoon and surface representation of the LotA DUB1 domain bound to the proximal ubiquitin (S1′ site) and the distal ubiquitin (S1 site). *B*, close-up view of the isopeptide bond formed between the catalytic cysteine residue C13 residue of DUB1 domain and the residue G75 from the C-terminal tail of Ub-PA. *C*, previously published structures OTU DUBs in complex with diUb of different linkage type. *Left*: structure of Cezanne/OTUD7B OTU domain bound to a K11-linked diUb. *Right*: structure of OTULIN OTU domain (C129A mutant) in complex with M1-linked diUb. *D*, *in vitro* DUB assays demonstrating the cleavage specificity of the LotA DUB1 domain. DiUb proteins of various linkage types were incubated with purified WT LotA DUB1 domain at 37 °C for 1 h, after which the cleavage products were resolved by SDS-PAGE and visualized using silver staining. *E*, residue K6 of the proximal ubiquitin is positioned 4.9 Å away from the C-terminal tail of the distal ubiquitin, mimicking the K6-linked diUb. *F*, cartoon representation of apo K6-linked diUb (PDB: 3ZLZ) in closed conformation. The hydrophobic residues I36 and I44 are shown as sticks. DUB, deubiquitinase; OTU, ovarian tumor; PDB, Protein Data Bank.

Upon further inspection of the structure, we observed an isopeptide bond between the catalytic cysteine C13 in DUB1 domain and the tail of one Ub (colored *magenta*) (Fig. 2, *A* and *B*). The binding site for this Ub should correspond to the distal Ub-binding site S1 in representative OTU DUBs. The second Ubn (colored *yellow*) is instead bound within another DUB1 domain pocket of considerable size (over 800 Å^2^) (Fig. 2*A*). The C-terminal tail of the second Ub is exposed to solvent, that is, it is remote from the catalytic cleft and no isopeptide bond is formed between them. We therefore conclude that this pocket corresponds to the proximal Ub-binding site S1’ (Fig. 2*A*), which provides a plausible explanation for the presence of a Ub band in the results of the size-exclusion chromatography with LotA_1-542_–Ub-PA in Fig. S1*A*. The existence of the proximal Ub-binding site S1′ in DUB1 distinguishes LotA from other OTU DUBs, which generally possess only the distal Ub-binding site or the S1′ is too small to accommodate Ub (31). To date, the available structures of OTU DUBs possessing an S1′ site in PDB include only the K11 linkage-specific Cezanne (PDB ID: 5LRV) (8) and the M1 linkage-specific OTULIN/FAM105B (PDB ID: 3ZNZ) (Fig. 2*C*).

It is widely agreed that the Ub linkage specificity of DUBs is defined by the orientation of the proximally bound Ub with respect to the catalytic site (3). Our structure clearly shows that K6 of the proximal Ub is oriented toward the center of the DUB1 catalytic triad C13, H237, and D10 (Fig. 2*B*), whereas all other lysine residues are distant from the catalytic center. Moreover, close examination of the interface between proximally and distally bound Ub moieties reveals that residue G75 in the C-terminal tail of distal Ub is located only 4.9 Å from the residue K6 of the proximal Ub. Hence, the proximally and distally bound Ub mimic a K6-linked diUb. We further hypothesized that the strong preference of LotA toward K6-linked diUb is determined by the DUB1 domain. Indeed, DUB assays show that LotA DUB1 cleaves K6-diUb (Fig. 2*D*), which is also in agreement with the previous report that the C13S mutation abolishes the DUB activity of LotA toward K6-linked diUb (28).

Of note, the conformation of the LotA DUB1-bound K6-linked diUb adopts an extended conformation that lacks Ub-Ub interchain interactions (Fig. 2*E*). Such conformation deviates significantly both from that of K6-linked Ub chains alone, where the I44 of both distal and proximal Ub form a hydrophobic interface (Fig. 2*F*), and that of K6-linked diUb in complex with USP30, where I36 of the distal Ub forms hydrophobic interface with the palm of USP30 (32, 33). In contrast, residues I44 and I36 of the LotA-bound diUb are exposed to solvent. Further comparison with all available structures of DUBs in complex with diUb chains revealed that solvent-exposed I44 and I36 residues are the prominent feature of K6-linked diUb bound to LotA. Taken together, these results show that LotA possesses a unique S1′ site that enables the binding of proximal Ub to LotA, allowing accurate positioning of its residue K6 relative to the C terminus of the S1 site-bound distal Ub.

### Structural basis for recognition of proximal Ub by the LotA DUB1 domain

The S1′ site in the DUBs is supposed to orient the linkage-specific lysine or the N-terminal methionine toward the catalytic site to ensure the selective cleavage of that linkage type (4). However, although some OTU DUBs such as LotB (24, 26), wMelOTU (30), and OTUD3 (4) display catalytic activity toward K6-linked diUb, no other known OTU DUB exclusively hydrolyzes K6-linked Ub chains, and no structure of an OTU DUB in complex with K6-linked diUb has been reported to date.

**Figure 3:**
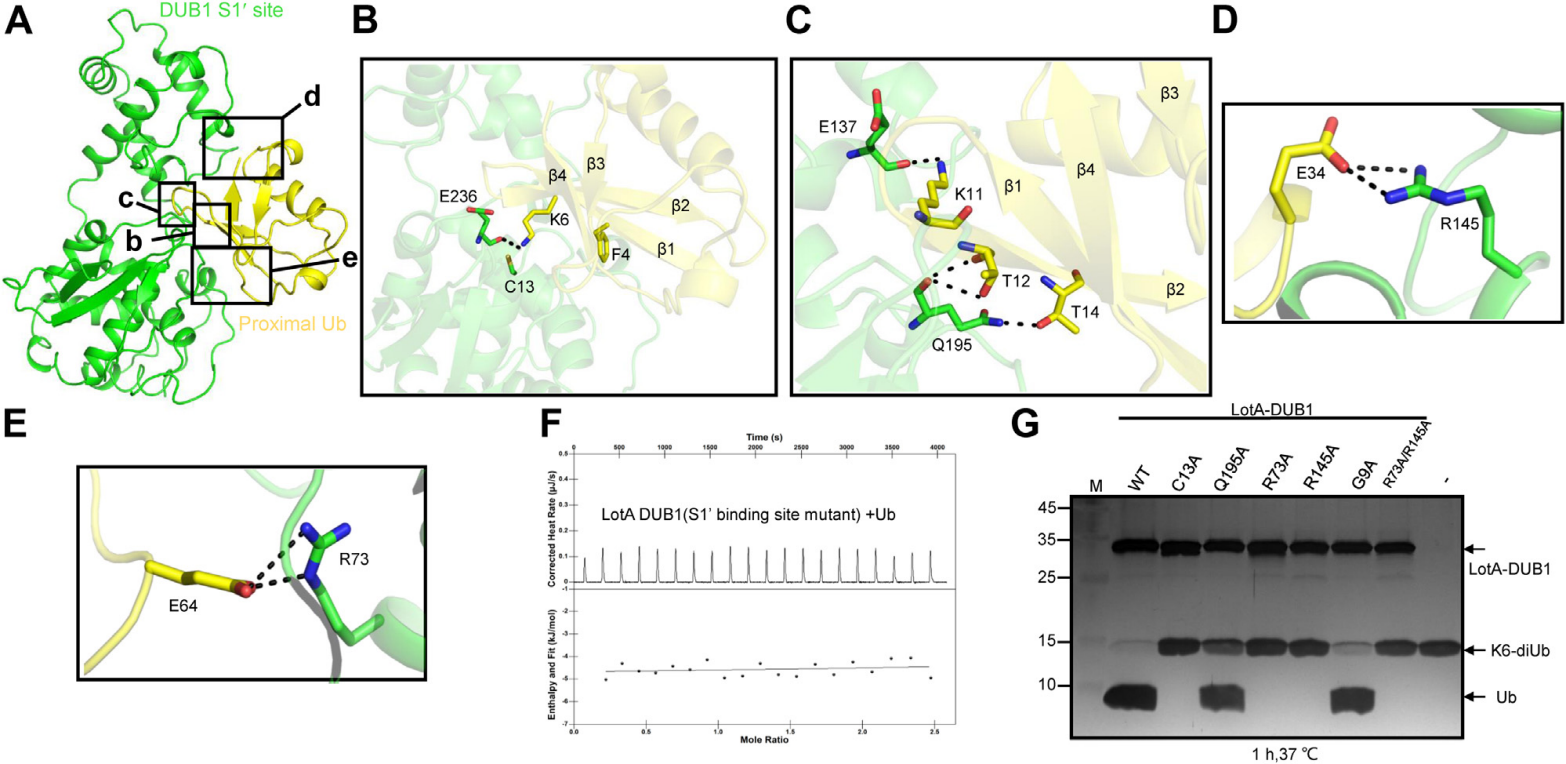
**Mechanism of proximal ubiquitin recognition by the DUB1 domain of LotA.***A*, cartoon representation of the LotA DUB1 domain bound to the proximal Ub. *B*–*E*, close-up views of the interactions between DUB1 domain and the proximal ubiquitin. Residues involved in interactions are shown as sticks and hydrogen bonds are shown as *black* dotted lines. Residues from the strands β1 and β2 of ubiquitin and residues R73 and R145 form key interactions with the DUB1 domain. The residue F4 of Ub, which forms important hydrophobic interactions with the palm subdomain of USP30, does not participate in interactions with the DUB1 domain of LotA. *F*, binding of LotA mutant LotA1-542 (R73, E137, R145, Q195, and E236 to Ala) to 6xHis Ub monitored by ITC. *G*, DUB assays *in vitro* of WT LotA and the variants with mutation of residues involved in the proximal ubiquitin binding, each LotA or the mutants was mixed with K6-diUb at 37 °C for 1 h. DUB, deubiquitinase; ITC, isothermal titration calorimetry.

Analysis of the interactions between the DUB1 domain and the proximally bound Ub reveals a recognition mechanism that strongly differs from the recognition of distal Ub by the OTU DUBs. The hydrophobic interactions mediated by the Ub residue I44, which are important for binding of distal Ub by OTU DUBs (4), are not formed between the proximal Ub and LotA. Meanwhile, the hydrophobic interactions between F4 on the strand β1 of Ub and the hydrophobic patch of the palm subdomain, which contribute to the recognition of proximal Ub by USP30, are not observed in LotA (32, 33). In the present structure, three distinct interacting regions between LotA DUB1 and proximal Ub that altogether account for 690 Å^2^ of buried surface area can be discerned (Fig. 3*A*). The LotA-interacting surface of the proximal Ub is composed of strands β1 and β2, helix α1, and the α3–β4 loop (Fig. 3*B*). The contacts are maintained mainly through hydrogen bonding, including those formed between K6 on the strand β1 and E236 of LotA, T12 and T14 on the strand β2 and Q195 of LotA, and K11 on the strand β2 and E137 of LotA (Fig. 3*C*). Additional hydrogen bonds are formed between E34 from the helix α1 and R145 of LotA (Fig. 3*D*), as well as E64 in the α2–β4 loop and R73 of LotA (Fig. 3*E*). When the aforementioned residues of LotA_1-542_ (R73, E137, R145, Q195, and E236) were simultaneously substituted to Ala, the binding affinity between LotA_1-542_ and 6xHis Ub were completely abolished (Fig. 3*F*), confirming their role for the proximal Ub covalent binding to the S1′ site of LotA_1-542_ (Fig. 1*D*).

To confirm the roles of these interactions between LotA and the proximal Ub in the cleavage of K6-linked Ub chains, we mutated the interacting LotA residues to alanine. The results of DUB assay indicate that the mutation Q195A decreases cleavage of K6-linked diUb, whereas the mutations R73A and R145A completely abolish the cleavage activity (Fig. 3*G*), confirming the critical importance of these residues for Ub recognition.

### Structural basis for recognition of distal Ub by the LotA DUB1 domain

**Figure 4:**
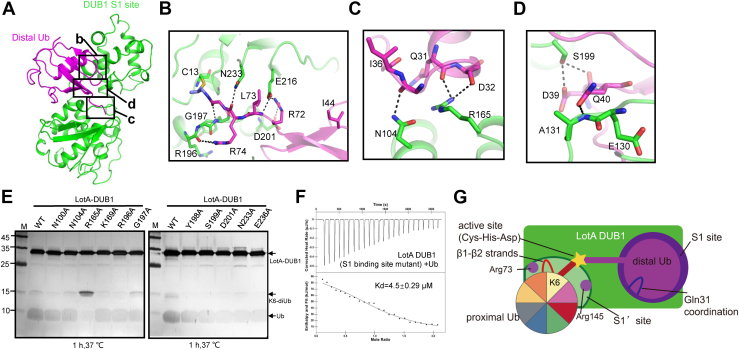
**Mechanism of distal ubiquitin recognition by the LotA DUB1 domain differs from typical OTU DUBs.***A*, cartoon representation of the LotA DUB1 domain bound to the distal Ub. *B*–*D*, close-up views of interactions between the DUB1 domain and the distal ubiquitin. Residues involved in interactions are shown as sticks and hydrogen bonds are shown as *black* dotted lines. The hydrophobic residue I44, which is typically important for interaction with OTU DUBs, does not form any contacts with the DUB1 domain of LotA. *E*, SDS-PAGE gel demonstrating the results of DUB assays performed with WT LotA and the variants with mutations of residues involved in distal ubiquitin binding, LotA or the mutants was reacted with K6-diUb at 37 °C for 1 h. *F*, ITC-based measurement of the binding affinity of LotA1-542 carrying mutation on the S1′ DUB binding site with 6xHis Ub. *G*, the mechanism for the specific recognition of K6-linked diubiquitin by LotA is significantly different from the mechanism for recognition of K6-linked diubiquitin by USP30. DUB, deubiquitinase; ITC, isothermal titration calorimetry; OTU, ovarian tumor.

Like in other cysteine-dependent DUBs, the C-terminal tail of the distal Ub is inserted into the catalytic cleft of the DUB1 domain and forms an isopeptide bond with the catalytic cysteine C13 (Fig. 4*A*). The distal Ub is bound to the DUB1 domain *via* three distinct regions with a total buried surface area of 960 Å^2^. In the first region, binding of the C-terminal tail of Ub is mediated by a network of hydrogen bonds. Residues R72 and R74 of the distal Ub, which typically participate in interactions between distal Ub and DUBs (34), are also involved in the hydrogen bonding with the DUB1 domain of LotA. However, the hydrophobic residue L73 that commonly forms hydrophobic interactions with DUBs (31) is exposed to the solvent. The side chain and backbone of R74 form hydrogen bonds with G197 and N233, respectively. R72 is stabilized by D201 and E216 of LotA (Fig. 4*B*). In the second region, which involves main chains of residues from the helix α1 of Ub, hydrogen bonds are formed between Q31 and D32 of Ub and R165 of LotA and between I36 of Ub and N104 of LotA (Fig. 4*C*). The third region includes hydrogen bonds between D39 and Q40 from the 3_10_ helix of Ub and S199 and E130 of LotA, respectively (Fig. 4*D*). It is noteworthy to mention that I44 and β1-β2 loop of Ub, which typically participate in interactions between distal Ub and DUBs (4), are not engaged in binding of the distal Ub to the LotA DUB1 domain.

To verify the role of these interactions in the binding of distal Ub to LotA, each of the aforementioned LotA residues was substituted to alanine, and the mutants were used to perform to *in vitro* DUB assays (Fig. 4*E*). The assay results indicate that the R165A mutant displays significantly reduced K6-linked diUb cleavage activity. In addition, we create a LotA_1-542_ mutant that all the residues of the S1 site were substituted to Ala, which was used to perform ITC assay to the 6xHis Ub (Fig. 4*F*), and the result shows that the affinity is comparable to the WT LotA_1-542_ (4.5 μM vs 3 μM), further confirming that binding affinity between LotA and 6xHis Ub measured by ITC (Fig. 1*D*) is due to noncovalent binding of Ub. Taken together, structural and experimental data clearly demonstrate that the distal Ub is recognized by the LotA DUB1 domain *via* a mechanism that significantly differs from analogous mechanisms of other OTU DUBs such as the recognition of K6-linked diUb by USP30 (Figs. 4*G* and S2*A*).

### The extra helix in LotA DUB2 is required for Ub recognition

**Figure 5:**
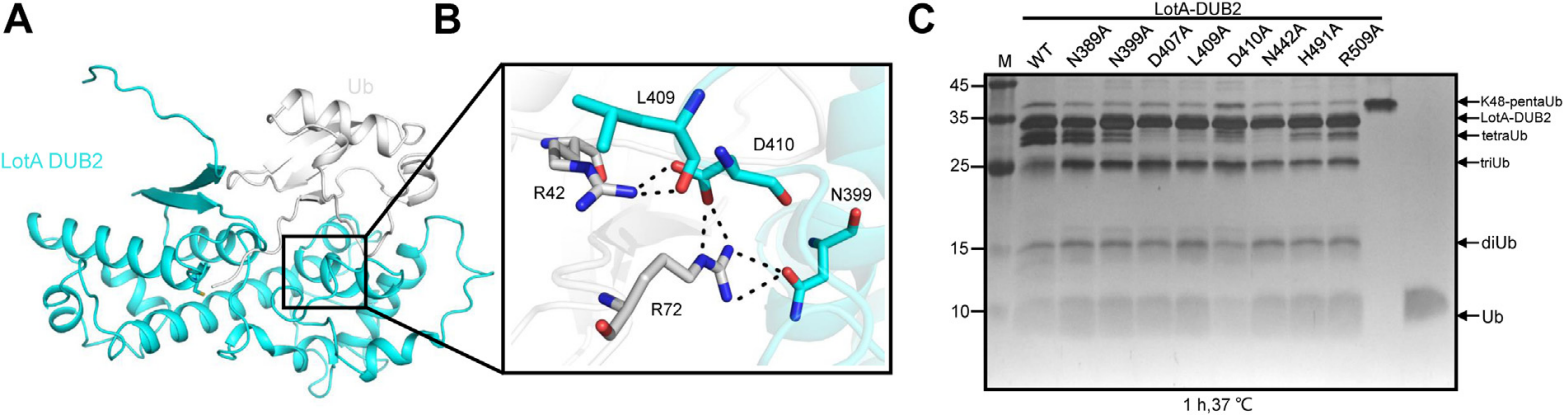
**The mechanism of distal ubiquitin (Ub) recognition by the LotA DUB2 domain.***A*, molecular docking of a Ub into the structure of the LotA DUB2 domain. *B*, close-up view of the interactions between the LotA DUB2 domain and the distally bound Ub. *C*, cleavage of K48-linked pentaUb by LotA DUB2 domain mutants. LotA DUB2 and K48-linked pentaUb were incubated at 37 °C for 1 h; note that the DUB2 mutant D410A exhibits a decrease in DUB activity against K48-linked pentaUb. DUB, deubiquitinase.

Although the distal Ub that should be bound to the LotA DUB2 domain is absent in the present structure, previous studies reported that DUB2 can react with Ub-PA and that the domain plays an important role in the cleavage of the polyUb chains (28). The DUB2 domain has an extended structure with an extra α-helix (residues D407–R413) that is similar to LotB and LotC but different from the typical globular papain-like fold of OTU DUBs (Fig. 5*A*). Docking of Ub onto the DUB2 domain suggests that the C-terminal tail of Ub is wrapped by the extra helix (Figs. 5*B* and S3). To investigate whether the extra helix is required for Ub recognition, we generated single point mutants D407A, L409A, and D410A and tested their cleavage activity using pentaUb. The D410A mutant displayed reduced pentaUb cleavage activity (Fig. 5*C*), which is in agreement with recently reported results (Fig. S2*B*) (35). Altogether, these findings indicate that the extra helix of the LotA DUB2 domain is required for Ub recognition.

## Discussion

Numerous bacterial effector proteins function as the E3 ligases or DUBs, reflecting the importance of manipulating host Ub pathways for pathogen infection (11, 14, 36). Previous studies discovered four OTU family DUBs were utilized by *L. pneumophila* for the regulation of the Ub network within host cells. Among these four OTU family DUBs, LotB preferentially cleaves K63-linked Ub chains (27) and LotC has a preference for Ub chains with K6, K11, and K48 linkage (24, 26). Structural studies of LotB and LotC revealed that the extra helices were important for distal Ub binding, but the molecular mechanism for the Ub chain selectivity is not fully understood (24, 26, 27). LotA is the first identified OTU DUB with two predicted DUB domains and the first identified OTU DUB that exclusively hydrolyzes K6-linked diUb (28).

OTU DUBs share a conserved fold, which upon hydrolysis of diUb requires the accommodation of distal Ub within the S1 site so that its C-terminal tail is positioned within the catalytic site. Thus, the orientation of distal Ub is supposed to be identical in each diUb molecule regardless of linkage type and the linkage specificity is determined by the proximal Ub that binds to the S1′ site, which provides a lysine residue for the formation of K6-linked chain. To date, four factors influencing the preference for a certain Ub linkage specificity of OTU DUBs have been identified (4). These factors include the presence of an additional Ub-binding domain (UBD), the ubiquitinated sequence in the substrate, as well as the presence of Ub-binding sites S1′ and S2 (4). Position and orientation of the proximal Ub in the S1′ site have also been demonstrated to play important roles in the Ub recognition. For example, the S1′ site of the pathogen *Orientia tsutsugamushi* DUB (otDUB) determines the chain specificity and the unique UBD can modulate DUB activity by sequestering the K48 diUb away from the activate site (36). Likewise, the difference in the Ub chains specificity of the LotA DUB1 domain and other OTU DUBs may depend on size of the S1′-binding site (27). The Ub-binding surface of the S1′ in OTU DUBs is negligible; to the best of our knowledge, LotA is the first characterized OTU DUB with an S1′ site whose surface area is sufficiently large to accommodate the proximal Ub high affinity.

Structural analysis of interactions between OTU DUBs and Ub has been typically performed with covalently modified Ub derivatives to generate stable enzyme–substrate complexes. While the mechanisms for recognition of the distal Ub by the cysteine-dependent DUBs have been extensively studied, to the best of our knowledge the structure of the LotA_1-542_–Ub-PA complex represents the first crystal structure of an OTU DUB in complex with K6-linked diUb. The present structure of LotA in complex with free Ub unexpectedly led to discovery of the additional Ub-binding site S1′ and allowed deeper insight into DUB specificity and mechanism of Ub binding. LotA DUB1 domain exclusively cleaves K6-linked diUb (28) and unlike in other OTU DUBs, we found that binding of the proximal Ub to the S1′ site of DUB1 helps position such chains for cleavage, so that the free Ub in the S1′ site is specifically bound to LotA DUB1 and the C-terminal tail of distally bound Ub is positioned in the vicinity of the K6 of Ub in the S1′ site, thus mimicking a K6-linked diUb. Hence, the structure presented in this study represents the first structure of an OTU family DUB that exclusively cleaves K6-linked diUb. Besides LotA, the CE family DUB, otDUB, from the pathogen *O. tsutsugamushi* was documented to bind free Ub moieties within the S2 site in addition to the active site S1. Analogously to the LotA DUB1 domain, the C-terminal tail of the Ub accommodated within the S2 site was positioned in the vicinity of K63 of the S1 site bound, thereby representing the K63-linked diUb (36).

Thus far, the mechanism for recognition of K6-linked polyUb chain has been studied for USP30 (32, 33), indicating that the hydrophobic interactions between the proximal Ub and USP30 play crucial role in the K6-linked polyUb chain recognition. Notably, the central F4 residue of Ub forms a hydrophobic patch with the palm domain of USP30 (32, 33). Moreover, residue I44 of both distal and proximal Ub forms an important hydrophobic interface with the palm domain of USP30 for its binding. Compared to USP30, our structure suggests that the proximal Ub is recognized by the LotA DUB1 domain in a distinct manner since the residue F4 of the proximal Ub and the residue I44 of proximal and distal Ub are all exposed to solvent.

The strong preference for cleaving longer Ub chains has previously been reported for the MINDY family DUBs MINDY1/2 (37) and ZUFSP/ZUP1 families of DUBs (38) but not for OTU DUBs. Both MINDY1/2 and ZUSFP DUBs possess multiple UBDs for polyUb chain cleavage. A recent study reported that the catalytic domain of MINDY1/2 has five distinct Ub-binding sites (46), suggesting the dependence of polyUb cleavage on chain length. Similarly, the DUB2 domain of LotA was found to possess multiple potential Ub- binding sites (Fig. S2*C*). However, how LotA DUB2 binds longer Ub chains and how they are hydrolyzed remains elusive.

In summary, in this study, we determined the structure of LotA_1-542_ with Ub and uncovered the unique DUB from the obligate intracellular pathogen *L. pneumophila* that is capable of specifically cleaving K6-linked diUb and polyUb chains relying on two OTU DUB domains. The LotA DUB1 folds into a unique structure with a large S1′ Ub-binding site to the proximal Ub to ensure its preference for the K6-linked diUb. In addition, the findings on LotA DUB2 presented here and a recently published study reveals multiple Ub-binding sites for longer Ub chains. Altogether, we present the first crystal structure of the tandem LotA DUB domains in complex with K6-linked diUb and advance the understanding of the mechanisms underlying its specificity for K6-linked Ub chains and dual DUB activity.

## Experimental procedures

### Protein expression and purification

ORFs encoding full-length LotA or its truncation variants including LotA_1–542_ were inserted into pET21a to generate plasmids for the expression of LotA or its truncation variants with N-terminal 6×His tag. All constructs were expressed in *E. coli* BL21(DE3) cells that were at the end of the expression collected by centrifugation. Collected cells were resuspended in a buffer containing 50 mM Tris–HCl (pH 8.0) and 150 mM NaCl and were then lysed by ultrasonication. After centrifugation at 17,000*g* for 30 min, LotA and its truncation mutants were purified using Ni^2+^-NTA column (Qiagen). After washing with a buffer containing 50 mM Tris–HCl (pH 8.0), 150 mM NaCl, and 20 mM imidazole, the target protein was eluted using the same buffer supplemented with a linear gradient of 50 to 250 mM imidazole. Fractions containing the target protein were pooled, concentrated to 0.5 ml, and loaded onto a Superdex 200 increase column (GE Healthcare) equilibrated with buffer containing 20 mM Tris–HCl (pH 8.0) and 150 mM NaCl for further purification.

Selenomethionine-labeled LotA_1–542_ was expressed in M9 medium supplemented with 2 mM MgSO_4_, 0.1 mM CaCl_2_, 0.5% w/v glucose, 2 mg/L biotin, 2 mg/L thiamine, and 0.03 mg/L FeSO_4_. At an *A*_600_ value of 0.5, 100 mg/ml of phenylalanine, lysine, and threonine, 50 mg/ml of isoleucine, leucine, and valine, as well as 80 mg/ml of selenomethionine (Chemie Brunschwig) were added to the cultures, which were then incubated for another 30 min. Expression was induced with 0.2 mM IPTG, and cells were further incubated at 16  °C on a shaker for 16 h. Cells were harvested at 5000*g* and 4  °C for 15 min, and pellets were resuspended in the lysis buffer (50 mM Tris (pH 8.0), 150 mM NaCl, and 5 mM β-mercaptoethanol), following which the protein was purified as described previously.

### Protein crystallization and data collection

To obtain the crystals of the LotA_1–542_–Ub-PA complex, the purified LotA_1-542_ was incubated with Ub-PA (UbiQ) at a 1:3 M ratio at 4 °C for 30 min. The mixture was afterward loaded onto a Superdex 200 increase column (GE Healthcare) to purify the LotA_1-542_–Ub-PA conjugate, which was then concentrated using an Amicon Ultra 30K centrifugal filter (4000*g*, 4  °C) to approximately 15 mg/ml.

For crystallization, the concentrated LotA_1–542_–Ub-PA complex was mixed with the reservoir solution at an equal volume and crystallized using the sitting drop vapor diffusion method at 16  °C. Crystals of the complex were obtained within 3 days in the condition containing 100 mM magnesium formate and 15% (w/v) PEG 3350. Diffraction quality crystals of the selenomethionine-labeled LotA_1–542_–Ub-PA complex were grown in the presence of 100 mM magnesium formate and 5% (w/v) PEG 3350. Crystals were cryopreserved in 20% (v/v) ethylene glycol before flash freezing in liquid nitrogen.

### ITC

ITC experiments were carried out using the Nano ITC low volume (TA instruments). All samples used in the assay were prepared in the buffer containing 20 mM Tris–HCl (pH 8.0) and 150 mM NaCl. Except for the titrant or titrate, all other components of the solution were adjusted to the same levels before titration. Typically, Ub (200–500 μM) in a syringe was titrated into a sample cell containing LotA (10–20 μM) following the multiple injections method. The experiment was performed at 25 °C. Obtained data were integrated, corrected, and analyzed using the NanoAnalyze software (TA Instruments) according to the single site–binding model.

### *In vitro* DUB assays

For reactions of LotA with Ub-PA (Boston Biochem), diUb, or K48-linked pentaUb, 1 μM Ub-PA or K48-linked pentaUb was mixed with 1 μM LotA in 20 μl DUB buffer (50 mM Tris (pH 7.5), 50 mM NaCl, 5 mM DTT) and incubated at 37 °C for 1 h. The reaction was stopped by adding 5 μl of 5×SDS sample buffer and boiling the sample for 5 min. The samples were then resolved by SDS-PAGE and visualized using silver staining.

### Analytic ultracentrifugation

Sedimentation velocity experiments were used to assess the molecular mass of LotA at 20 °C on a Beckman XL-A analytical ultracentrifuge equipped with absorbance optics and An-60 Ti rotor (Beckman Coulter). LotA_1-542_ was diluted to an absorbance at 280 nm of 1 in a 1.2 cm path length and the rotor speed was set to 60,000*g* for all samples. Differential sedimentation coefficient c(s), frictional coefficients, and molecular mass were calculated using SEDFIT software (https://sedfitsedphat.github.io/).

### Structure determination and refinement

Crystal diffraction data for the LotA_1-542_–Ub-PA complex were collected at the Shanghai Synchrotron Radiation Facility (SSRF) BL-02U1 using the SAD) method. Datasets were processed on site with the HKL-2000 package. Autosol program of PHENIX package (39) was used for SAD phasing, followed by iterative manual building using Coot (40) and refinement in PHENIX. The crystals belong to space group P 2_1_ 2_1_ 2_1_ with unit cell dimensions of a = 66.75 Å, b = 118.95 Å, and c = 84.28 Å. The final structure was refined to 2.64 Å resolution (R-value and R-free of 21.43% and 25.52%, respectively) (Table 1). Structure quality was analyzed during PHENIX refinements and later validated in the PDB validation server (41). Structural figures were generated using PyMol (Schrödinger, LLC).

### Computational modeling

To uncover how DUB2 domain of LotA binds distal Ub (PDB ID: 2AYO), we used the rigid protein-protein docking software ZDOCK (version 3.03; https://sedfitsedphat.github.io/) to generate a large amount of binding conformations (42). The binding conformations were clustered and screened by searching for the ones with a cysteine residue at the interface because the Ub is assumed to form a disulfide bond with its target. After screening, six binding conformations near the catalytic residue C303 of DUB2 domain remained. To remove the atomic clashes between LotA and Ub, side chain repacking was carried out and 1 ns molecular dynamics simulation was performed (43). Finally, the binding conformation with the lowest energy was considered as the model of DUB2–distal Ub complex structure.

## Data availability

Coordinates and structure factors for the LotA1-542–Ub-PA complex have been deposited in the Protein Data Bank under the accession code 7W54.

## Supporting information

This article contains supporting information.

## Conflict of interest

The authors declare that they have no conflicts of interest with the contents of this article.

## Supporting information

Fig S3

LotA

LotA
