# Supplementary material for: Structural basis for the dual catalytic activity of the Legionella pneumophila ovarian tumor (OTU) domain deubiquitinase LotA
Source: J Biol Chem. 2022 Aug 22;298(10):102414. doi: 10.1016/j.jbc.2022.102414 (PMC9486567; doi:10.1016/j.jbc.2022.102414)
Supplement: Supporting information [file mmc1.docx]

**Figure S1**

The overall structure of LotA_1-542_-Ub-PA **(a)** The size-exclusion chromatography analysis of LotA_1-542_-Ub-PA and LotA_1-542_, respectively. **(b)** Two molecules of LotA_1-542_ and four molecules of Ub are present in a crystal asymmetric unit (ASU), with two molecules of Ub bound by each molecule of LotA_1-542_. The LotA_1-542_-Ub-PA complex analyzed in this study is color-coded as in Figure 1E. **(c-d)** The two LotA_1-542_ molecules within the ASU exhibit obvious conformational differences, with a core root mean square deviation (RMSD) of 0.826 Å, but the DUB1 and DUB2 domains in each LotA_1-542_ are display a greater degree of similarity to each other, with a RMSD of 0.580 Å and 0.507 Å, respectively. **(e)** Results of AUC demonstrating that LotA_1-542_ exhibits a molecular mass of 62 kDa in solution (i.e., exists as a monomer in solution).

**Figure S2**

The comparation of the K6-linked diUb recognition between LotA and USP30. **(a)** The model for specific recognition of the K6-linked diUb by USP30. **(b)** Superimposition of the structure of LotA DUB2 domain from this study with the recently released structure of LotA DUB2 domain (PDB:7F9X). **(c)** Multiple potential ubiquitin binding sites within the DUB2 domain of LotA.

**Figure S3**

Structural basis for the distal Ub binding of LotA DUB2. Docking of ubiquitin into the structure of the LotA DUB2 domain (left panel). Close-up views display the interactions between the DUB2 domain and the distal Ub (inset panel).
