# Supplementary material for: Structural basis for the dual catalytic activity of the Legionella pneumophila ovarian tumor (OTU) domain deubiquitinase LotA
Source: J Biol Chem. 2022 Aug 22;298(10):102414. doi: 10.1016/j.jbc.2022.102414 (PMC9486567; doi:10.1016/j.jbc.2022.102414)
Supplement: Table S1 [file mmc2.docx]

**Table S1.** Dali search results against PDB using the structure of LotA DUB1 domain (1–266 aa) as search query

| **No.** | **PDB ID-Chain No.** | **Z scores** | **RMSD (Å)** | **Identity (%)** | **Description** |
| --- | --- | --- | --- | --- | --- |
| 1 | 7BU0-A | 10.9 | 5.1 | 14 | The *L.pneumophila* deubiquitinase Lem27 in complex with Ub-PA |
| 2 | 6KS5-B | 9.5 | 4.3 | 14 | The *L.pneumophila* deubiquitinase Ceg23 |
| 3 | 4DDG-A | 8.1 | 3.5 | 15 | The human  OTUB1/UbcH5b~Ub/Ub |
| 4 | 6W9R-B | 7.5 | 3.0 | 15 | an OTU deubiquitinase from Wolbachia pipientis wMel bound to ubiquitin |
| 5 | 4BOU-A | 7.3 | 3.0 | 13 | Structure of OTUD3 OTU domain |
| 6 | 3PHU-B | 7.0 | 3.0 | 10 | OTU Domain of Crimean Congo Hemorrhagic Fever Virus |
| 7 | 3C0R-A | 6.5 | 2.7 | 19 | The OTUB1 Ovarian  Tumor (OTU) domain in  complex with Ubiquitin |
| 8 | 3TMP-E | 6.4 | 2.7 | 15 | The catalytic domain of human deubiquitinase DUBA in complex with ubiquitin aldehyde |
| 9 | 6SAK-A | 5.9 | 3.7 | 13 | Structure of the OTULINcat C129A - SNX27 PDZ domain complex |
