# Supplementary material for: Structural basis for the dual catalytic activity of the Legionella pneumophila ovarian tumor (OTU) domain deubiquitinase LotA
Source: J Biol Chem. 2022 Aug 22;298(10):102414. doi: 10.1016/j.jbc.2022.102414 (PMC9486567; doi:10.1016/j.jbc.2022.102414)
Supplement: Table S2 [file mmc3.docx]

**Table S2.** Dali search results against PDB using the structure of LotA DUB2 domain (284–520 aa) as search query

| **No.** | **PDB ID-Chain No.** | **Z scores** | **RMSD (Å)** | **Identity (%)** | **Description** |
| --- | --- | --- | --- | --- | --- |
| 1 | 7BU0-A | 9.5 | 4.2 | 17 | The *L.pneumophila* deubiquitinase Lem27 in complex with Ub-PA |
| 2 | 4DDG-A | 6.8 | 4.5 | 15 | The human  OTUB1/UbcH5b~Ub/Ub |
| 3 | 6W9R-B | 4.9 | 3.5 | 17 | an OTU deubiquitinase from Wolbachia pipientis wMel bound to ubiquitin |
| 4 | 6SAK-A | 4.6 | 3.6 | 9 | Structure of the OTULINcat C129A - SNX27 PDZ domain complex |
| 5 | 4BOU-A | 4.4 | 2.9 | 17 | Structure of OTUD3 OTU domain |
| 6 | 5XDA-B | 3.8 | 3.4 | 12 | Structural basis for Ufm1 recognition by Ufm1-specific protease (UfSP) |
| 7 | 3C0R-A | 3.7 | 3.4 | 16 | The OTUB1 Ovarian  Tumor (OTU) domain in  complex with Ubiquitin |
| 8 | 3PHU-B | 3.7 | 3.1 | 9 | OTU Domain of Crimean Congo Hemorrhagic Fever Virus |
| 9 | 3TMP-E | 3.7 | 2.9 | 16 | The catalytic domain of human deubiquitinase DUBA in complex with ubiquitin aldehyde |
