# Supplementary figures and images for: Structural basis for the dual catalytic activity of the Legionella pneumophila ovarian tumor (OTU) domain deubiquitinase LotA
Source: J Biol Chem. 2022 Aug 22;298(10):102414. doi: 10.1016/j.jbc.2022.102414 (PMC9486567; doi:10.1016/j.jbc.2022.102414)

**a**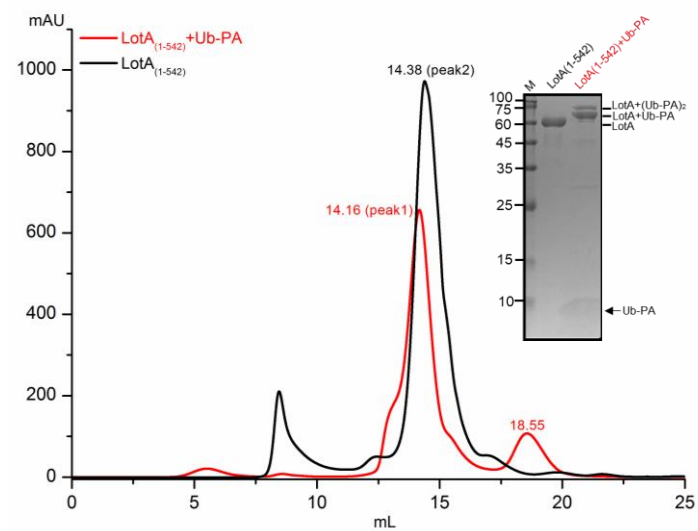**b**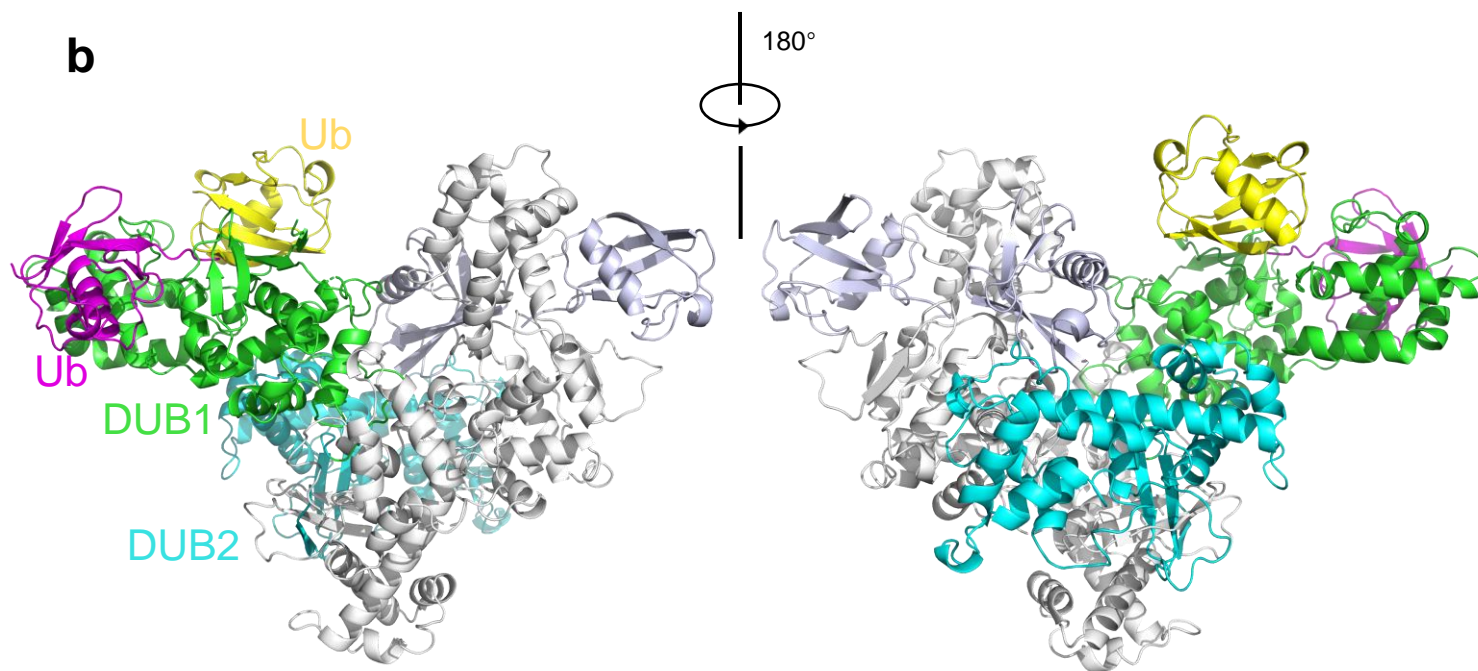**c**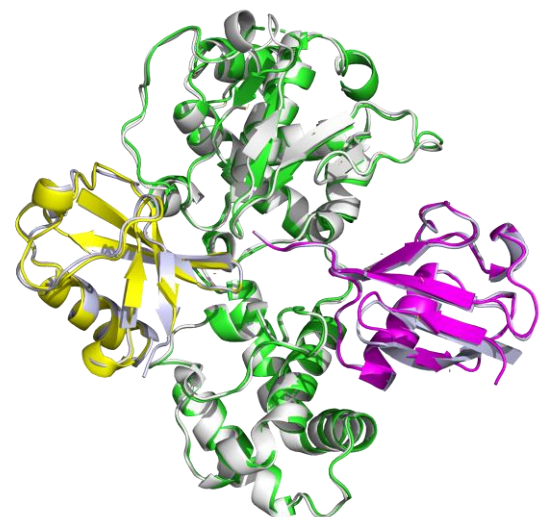

RMSD=0.580 Å

**d**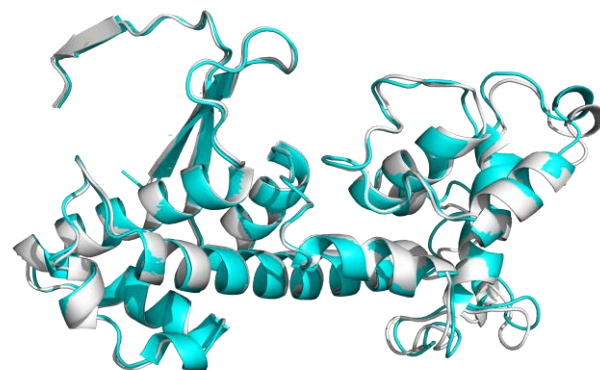

RMSD=0.507 Å

**e**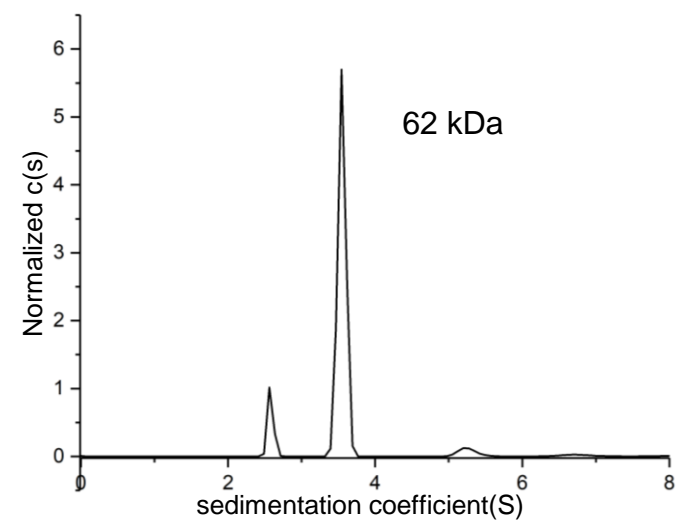

Supplement: Fig S1 [file mmc4.pdf]

**a**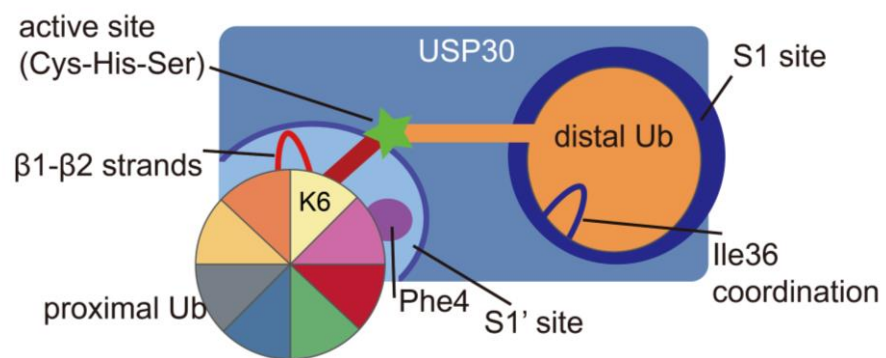**b**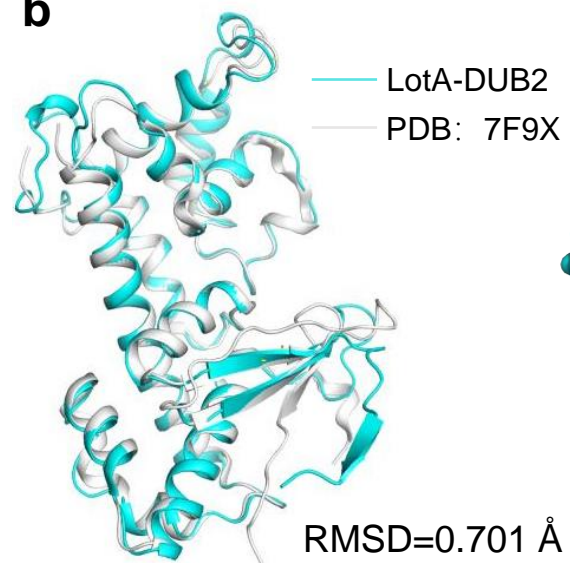**c**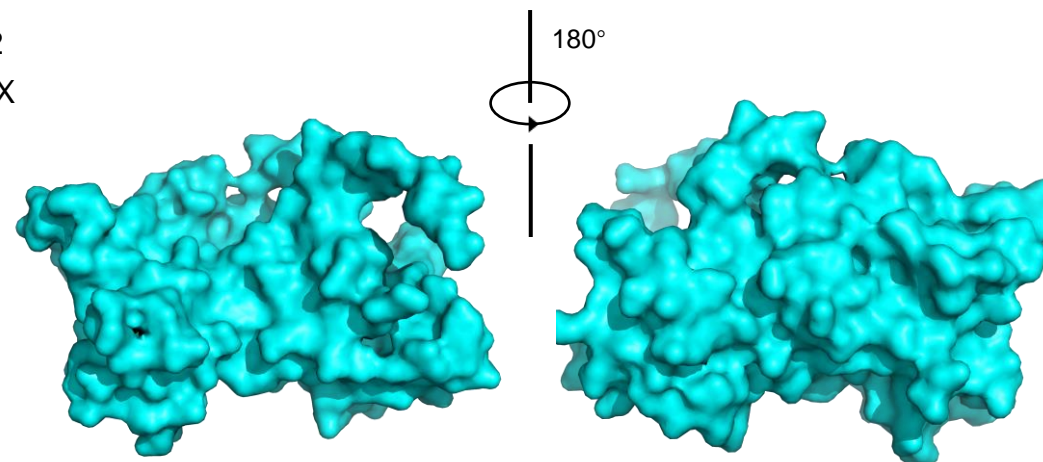

Supplement: Fig S2 [file mmc5.pdf]

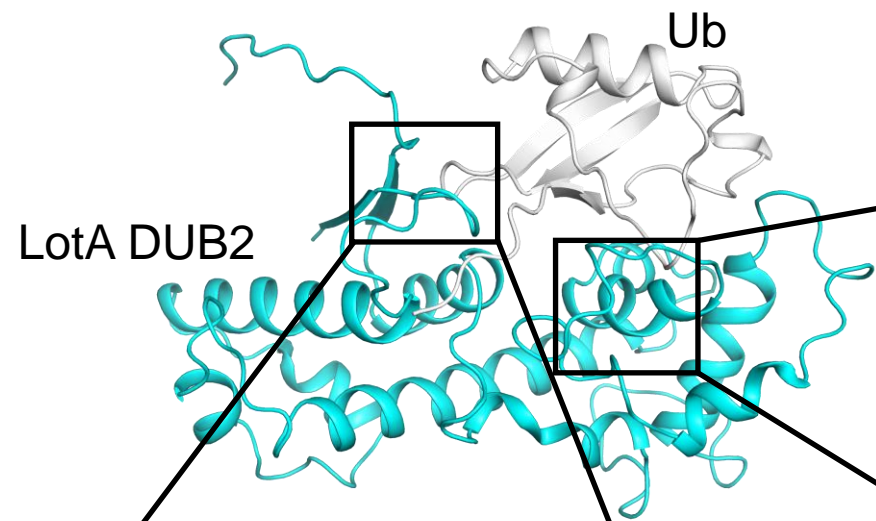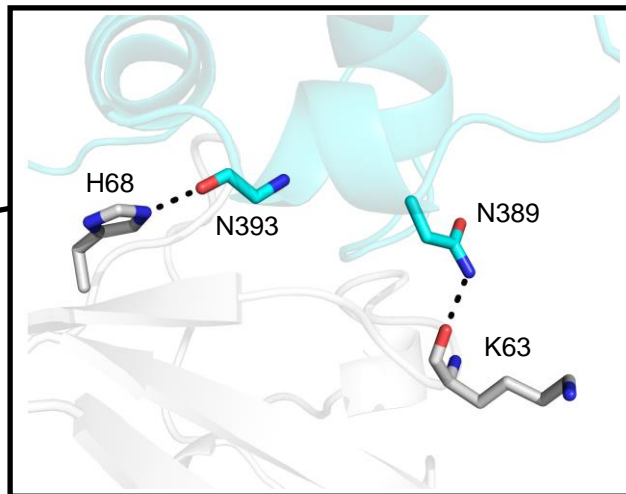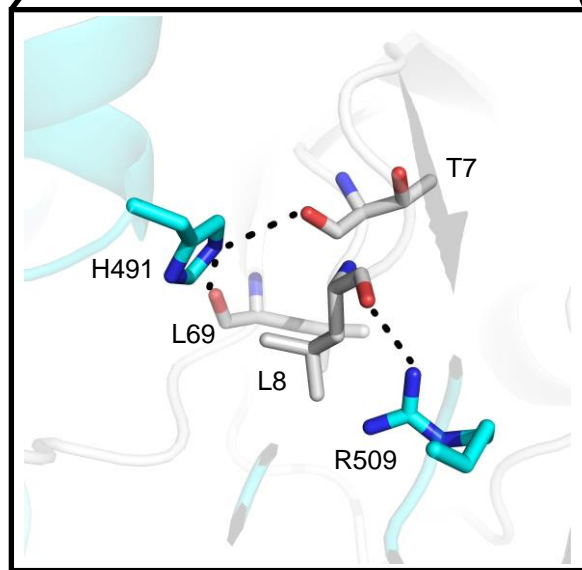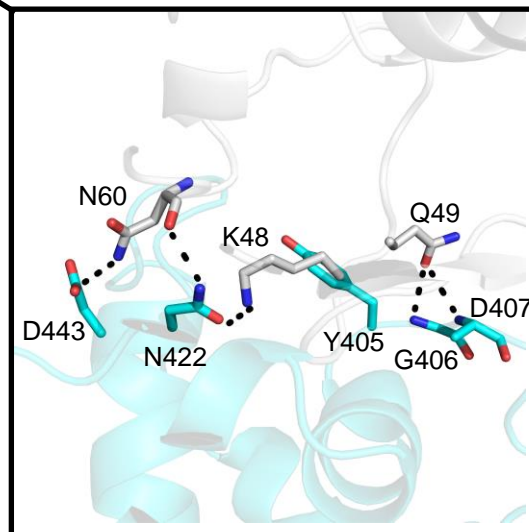

Supplement: Fig S3 [file mmc6.pdf]
